# Dr. Williams’s Criticism

**Published:** 1896-03

**Authors:** 


### DR. WILLIAMS'S CRITICISM.

It is, probably, quite as gratifying to an editor to find his articles antagonized as to receive commendation. In both cases it is quite evident the object of writing has been attained: that of impressing two classes of mind, the resulting effort being the eventual establishment of the disputed question upon a truthful foundation.

The editorial that has called forth this hurried criticism of Dr. Williams was not intended as a scientific essay; in fact, was not desired to be more than all editorials should be,—suggestive. The readers of journals are supposed to be intelligent and fully capable of doing their own thinking, and it is not for any editor to burden his special pages with tables of experiments or illustrations. All editorial writing must therefore be "extremely lame," as Dr. Williams phrases it.

We have not the space to reply to his somewhat dogmatic assertions, nor would we deem it necessary at this stage of the controversy. His premises may be, and probably are, based on actual investigations, and we wait with some impatience the production of these facts. It may, however, be said that our limited practical knowledge of microscopical work, extending not more than something over three decades, does not enable us to understand how Dr. Williams can prove by this method that the "decay of the teeth is not primarily dependent upon the exact amount of lime salts which they may contain." This is not made clear in his criticism.

It now belongs to him to show that Dr. Black's generalizations, founded on experiments, can all be sustained by the microscope. When this is done the readers of this journal will be better prepared to answer, in their own minds, without our aid, whether it is better to fill children's teeth from twelve to fifteen with gold or wait for years to give the increase of density demonstrated to take place by Dr. Black.

Will Dr. Williams, in his forthcoming articles, explain why a gold filling will devitalize a pulp in so-called soft teeth, and may not in what are termed dense teeth? Why in some teeth caries comes to a full stop, with the result of an ebonized surface (eburnated dentine)? Will he explain why the environments should destroy certain teeth so rapidly as to expose the basis-substance, and not affect certain other teeth in other mouths with, apparently, similar environments? Why pitted teeth rarely decay in the depressions? Why some teeth are so dense that they readily give off sparks, like flint, under the excavator? Why some children at twelve have pulp-nodules, and in others the dentine will cut like old cheese? These and many more problems connected with this subject await solution, and, while the writer has held and taught certain hypotheses regarding these conditions, we want facts. Can Dr. Williams, through the aid of the microscope, answer these interrogatories?

